# RUST: A Robust, User-Friendly Script Tool for Rapid Measurement of Rust Disease on Cereal Leaves

**DOI:** 10.3390/plants9091182

**Published:** 2020-09-11

**Authors:** Luis M. Gallego-Sánchez, Francisco J. Canales, Gracia Montilla-Bascón, Elena Prats

**Affiliations:** CSIC-Institute for Sustainable Agriculture, 14004 Cordoba, Spain; lmgallego@ias.csic.es (L.M.G.-S.); fjcanales@ias.csic.es (F.J.C.); gmontilla@ias.csic.es (G.M.-B.)

**Keywords:** rust, infection frequency, disease severity, image analysis, Fiji, ImageJ

## Abstract

Recently, phenotyping has become one of the main bottlenecks in plant breeding and fundamental plant science. This is particularly true for plant disease assessment, which has to deal with time-consuming evaluations and the subjectivity of visual assessments. In this work, we have developed an open source Robust, User-friendy Script Tool (RUST) for semi-automated evaluation of leaf rust diseases. RUST runs under the free Fiji imaging software (developed from ImageJ), which is a well-recognized software among the scientific community. The script enables the evaluation of leaf rust diseases using a color transformation tool and provides three different automation modes. The script opens images sequentially and records infection frequency (pustules per area) (semi-)automatically for high-throughput analysis. Furthermore, it can manage several scanned leaf segments in the same image, consecutively selecting the desired segments. The script has been validated with nearly 900 samples from 80 oat genotypes ranging from resistant to susceptible and from very light to heavily infected leaves showing a high accuracy with a Lin’s concordance correlation coefficient of 0.99. The analysis show a high repeatability as indicated by the low variation coefficients obtained when repeating the measurement of the same samples. The script also has optional steps for calibration and training to ensure accuracy, even in low-resolution images. This script can evaluate efficiently hundreds of leaves facilitating the screening of novel sources of resistance to this important cereal disease.

## 1. Introduction

Rust diseases are caused by a specialized group of fungi from the Pucciniales order, which are able to infect a diverse group of hosts, including ecologically and economically important crops and trees. Rust fungi are obligate biotroph parasites with intriguing and complex life cycles. This, together with their highly efficient spread mechanisms, make crop management practices inefficient for their control. Thus, the global prevalence of Pucciniales hinders production of many crops worldwide. Rust has been one of the most destructive cereal diseases since ancient times when, not without reason, the Romans created Robigus, the god of rust, to whom they appraised each spring in a ceremony to hold back his rage. Rust damages reduce the economic value of the grain, since infection affects the grain yield, kernel weight, and groat percentage [1,2,3]. Furthermore, crown rust infection weakens straw production and causes oat plants to lodge [4]. Throughout the 20th century, cereal rust epidemics have occurred intermittently in different regions of the world. They have been particularly damaging in Mediterranean environments where populations have been found to be more virulent [5].

Breeding for resistance is the most effective, economical, and environmentally-friendly mean to control rust [6,7]. However, it is a lengthy process that involves the identification of novel sources of resistance from large screenings [8]. Recent advances in genome and transcriptome sequencing have opened up great prospects to dissect the genetic determinants that underlie host infection processes and resistance mechanisms. However, the ability to translate knowledge of genomic variants into desired resistant phenotypes requires a more complete understanding of the genotype-phenotype relationship. Unfortunately, our ability to study plant phenomics has not progressed at the same rate as our ability to sequence genomes and transcriptomes. Therefore, evaluation of leaf rust diseases has become the main bottleneck to advance the breeding for resistance to these pathogenic fungi.

Proper evaluation of rust requires the understanding of its life cycle. Rust development in cereal hosts occurs during the uredial phase of the fungus [9]. This asexual phase involves repeated cycles of infection and sporulation that can be repeated every 2 weeks [10]. After deposition of urediniospores on the host, the spore germinates and germ tube grows until it contacts a stomata. Subsequently, an appressorium develops on the guard cells and a penetrating hypha penetrates through the stomata [11,12]. The penetrating hypha develops a substomatal vesicle from which a secondary hypha and a haustorium mother cell form at its tip. Upon contact between the haustorium mother cell and the mesophyll cell, the fungus develops an infection peg, which penetrates the mesophyll cell. Then, it forms a feeding structure, the haustorium, to absorbs nutrients for further fungal growth [13]. Intercellular growth of the infection hyphae continues until a fungal colony forms, giving rise to sporulating uredinia that produce a new set of urediniospores after 7–10 days. These urediniospore masses emerge through the rupture of the foliar epidermis as brownish-orange pustules, elliptical in shape, generally surrounded by a chlorotic halo [14,15,16,17]. Rust disease severity can be measured as percentage of diseased tissue as for other diseases. However, the most accurate method to score rust damages is to measure the infection frequency, that is, the number of pustules per unit square area. Visual assessment of disease severity is subjective and fairly imprecise as it is prone to operator bias (i.e., operator’s physical and/or working conditions) [18,19]. Evaluation of rust infection frequency is more accurate since counting pustules is less subjective than estimating disease severity but it is time-consuming and tedious.

In recent years, there has been considerable interest and progress in the development of digital image analysis systems for the assessment of plant diseases [20,21]. Through digital image analysis, it is possible to streamline processes and analyze information [21,22]. Visual light imaging of diseased leaves can be obtained with relatively common equipment such as a scanner (destructive imaging) or a camera (non-destructive imaging). Imaging-based systems allow the collection of hundreds of digital images per unit time and their analysis with a high degree of automation. Early work by Kokko et al. [23], Price et al. [24] or Kampmann and Hansen [25], with either black and white or color images, demonstrated that image analysis was superior to visual rating in different pathosystems including the coffee-leaf rust (*Hemileia vastatrix*) pathosystem. More recently, another image-based analysis system was able to identify and evaluate different classes of resistance to *Septoria tritici* blotch on wheat [26,27]. By contrast, some studies reported poor agreement between image analysis and rater estimates or actual values, including evaluation of the oat leaf rust (*Puccinia coronata* f. sp. *avenae*) on oats (*Avena sativa*) [28], although, the most recent work highlighted the potential of image analysis for plant disease evaluation (reviewed in [29]). Accordingly, great progress has been made in recent years with regard to disease assessment through digital image analysis. However, several pitfalls remain. Most of the available software evaluate disease severity as percentage of diseased tissue, and only a few allow counting the number of pustules per unit area (i.e., Assess [30]), which, as indicated above, is the most accurate parameter to estimate rust infection. In addition, most of the available software are expensive, such as the most widely used Assess [30] or Image-Pro Plus software [31], which are not affordable for small or more modest research groups. On the other hand, users of open source image analysis software, such as ImageJ/Fiji [32,33], often need computer programming skills and time to write macros [34] to adapt them to their needs.

In this work, we have developed the Robust, User-Friendly Script Tool (RUST), an image processing-based application to facilitate the efficient and accurate measurement of rust diseases in cereals (although it can be used in other crops). It has been implemented as a macro for Fiji (formerly ImageJ), which is an image processing package released as open source under the GNU General Public License that facilitates scientific analysis of images [33].

## 2. Results

### 2.1. Running the Script

The RUST script is implemented as a plug-in of Fiji software, which is open source and free to access at https://imagej.net/Fiji/Downloads. Before starting, the color transformer plug-in, available at https://imagej.nih.gov/ij/plugins/, must be installed in Fiji. Then, users just need to copy and paste the RUST.txt file (Supplementary material Script S2) into the “Macros>Toolsets” folder. This installs the RUST tool within your Fiji software. Additionally, users can download the ImageJ version 1.52p used to develop this script with the color transformed plug-in and RUST script installed at https://drive.google.com/file/d/1kdvva3Y0EfDMXxGI4kH3nKAMWzi_jM9i/view?usp=sharing. The RUST script can be started at any time by clicking “>>” button on the Fiji toolbar, selecting RUST, and pressing the key “S” (for Start). During evaluations, the script can be re-started at any time by pressing “E” (for Exit) and then “S” keys.

**Figure 1:**
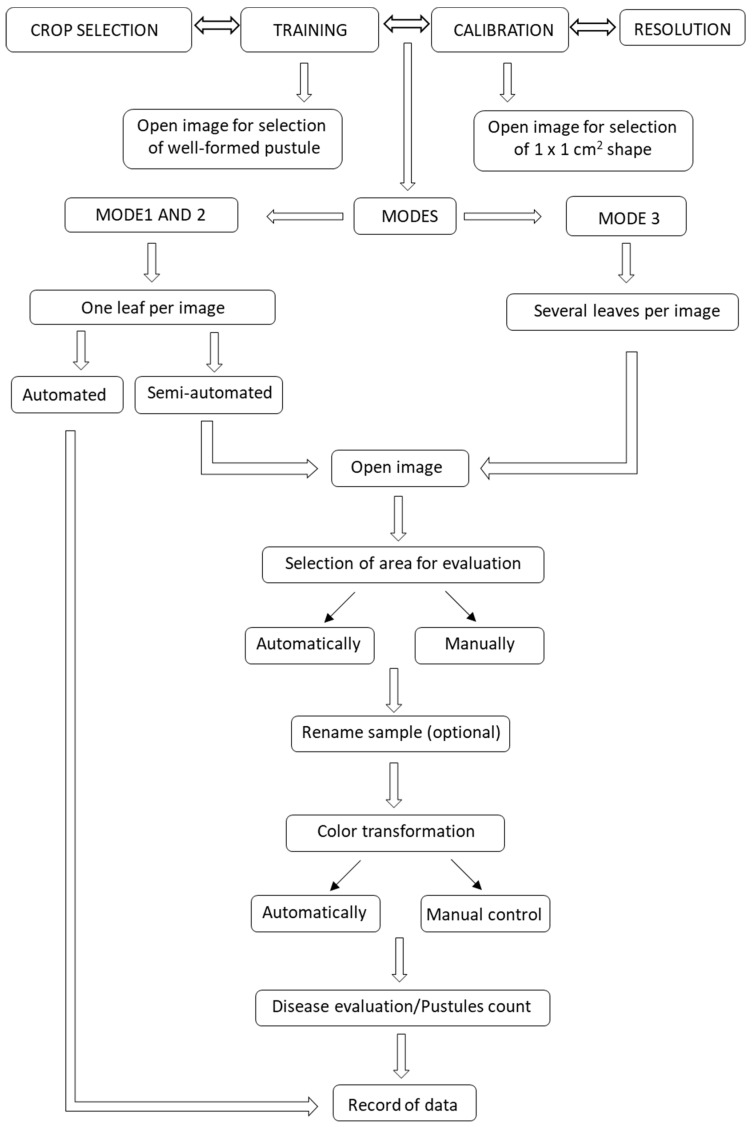
Scheme of the process followed by the Robust, User-Friendly Script Tool (RUST) to evaluate and record rust diseases.

**Figure 2:**
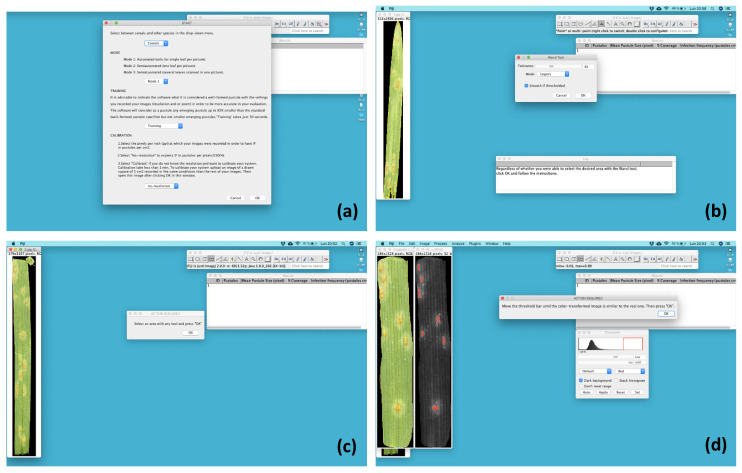
Screenshot of the Fiji application running RUST. (**a**). Screenshot for selecting crop, mode of evaluation, training/calibration option, and resolution. (**b**) and (**c**) Screenshot of the area selection step through the (**b**) automated wand tool or (**c**) manual selection. (**d**). Screenshot of the color transformation step that identifies rust pustules.

Figure 1 shows a diagram of the different steps through which RUST processes the data. After starting the script, a first window describes the tool and shows the instructions to access the next window where the user can define the evaluation settings (Figure 2a). There, users can select the targeted crop (cereals or other crops), the evaluation mode, and whether they want to perform the training and calibration steps. Users can also enter the image resolution if known. There are three evaluation modes (Figure 1). Mode 1 (see video in Supplementary Material S3) is the most automated mode. It collects the disease parameters on a set of different images without user intervention. This mode only works with images that contains a single leaf. In this mode, the analysis of 10 images takes approximately 30 s. Mode 2 and 3 (watch videos in Supplementary Materials S4 and S5, respectively) are semi-automatic, which means that users can control various steps of the evaluation to improve accuracy. Mode 2 only accepts files containing one leaf per image while mode 3 uses files containing multiple leaves scanned into one image. In both modes, users can control the steps of: (i) selection of the evaluated area by using the wand tool, a shape or the manual selection tools (Figure 2b,c); (ii) sample renaming, which can be particularly useful for images containing many scanned leaves; and finally (iii) color transformation, which allows users to adjust the transformed image to closely resemble the original (Figure 2d). In modes 2 and 3, the analysis of 10 images that require color adjustment and manual area selection, takes approximately 2 min.

During the area selection step (Figure 1), we observed that certain image characteristics, such a good color contrast between the healthy leaf, the background, and the pustule increase accuracy. For this reason, the area is better delineated when the pointer is located on the green part of the leaf and not within a pustule and on images with a black background. This optimal situation may not always be achieved. If the wand tool does not allow the precise delineation of the leaf edge, users can select one of the Fiji shape tools to manually select the area for evaluation. The rectangle or polygon tools are usually the most suitable tools for cereal leaves.

Following the color transformation step, the script estimates automatically the number of pustules, the average pustule size, and the percentage of diseased tissue and display it on a table that can be saved or exported to the spreadsheet (Figure 1).

**Figure 3:**
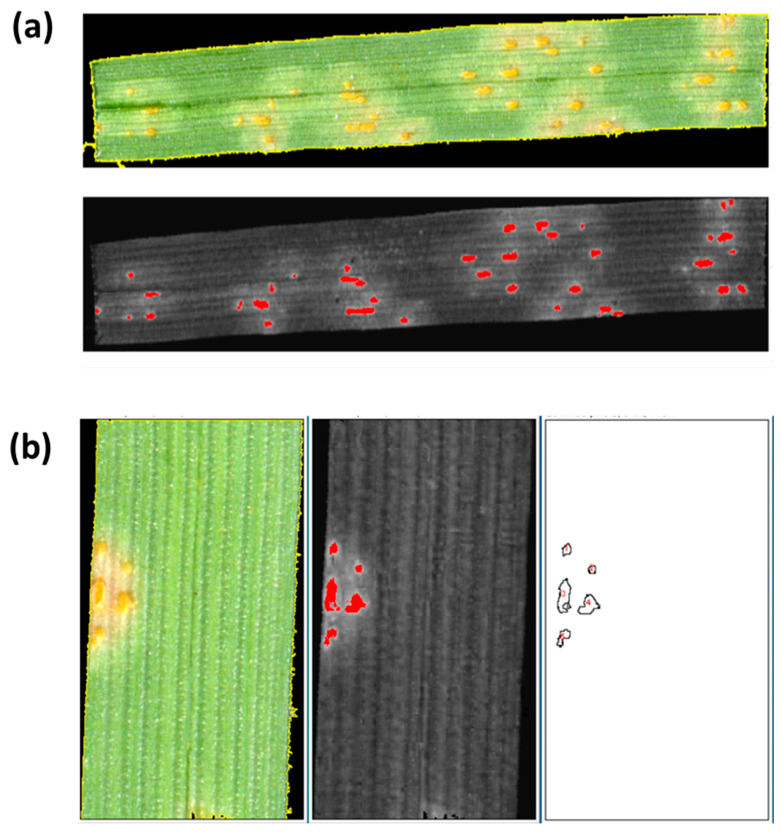
Discrimination of pustules from healthy tissue by RUST script. (**a**) Detection of primary rust pustules. (**b**) Detection and counting of a ring of secondary pustules surrounding a primary pustule.

The script was originally designed to evaluate rust diseases in cereals. However, other samples were also evaluated in order to test its applicability to other crops. To this aim, the script was implemented with the possibility to apply red projections during the color transformation step. This can increase accuracy in crops that may differ from cereals in the contrast between the green color of the leaf and the color of the pustules, widening the use of the script. These color transformation algorithms discriminated efficiently primary pustules from the healthy tissue (Figure 3a). Furthermore, under field conditions, rings of secondary pustules generally appear around the primary pustules. These rings of secondary pustules are produced by hyphae that grow across the intercellular spaces spreading radially from the primary pustule. Their abundance is associated with the fungus efficiency to produce secondary infections and/or post-haustorial resistance mechanisms that limit this efficiency. The RUST algorithm efficiently identifies and counts these smaller and closer secondary pustules when they are present (Figure 3b).

### 2.2. Validation of Script

**Figure 4:**
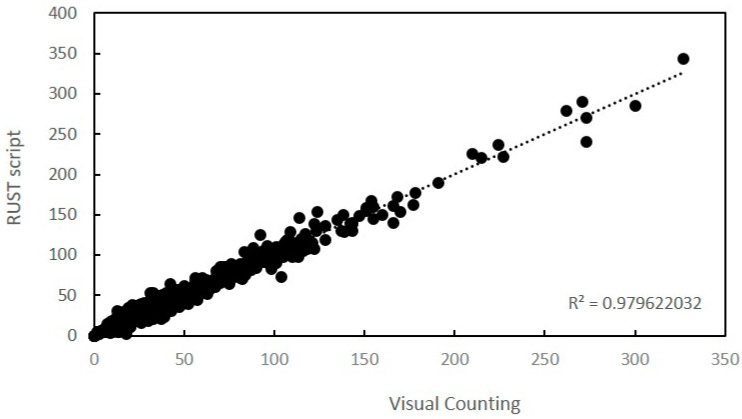
Linear relationship between the number of pustules estimated visually or with RUST.

In order to validate the accuracy of the script for the evaluation of rust diseases, 892 leaves were evaluated by visual pustule counting and digitally through the RUST script. We used a set of 80 different oat genotypes that varied in their susceptibility to rust and we used different inoculum densities to have a wide range of pustule density on the leaves. Bias, accuracy, precision, and agreement measures using Lin’s Concordance Correlation (LCC) and linear regression analysis for counts of pustules of rust on leaves indicates that the results obtained by visual counting and by the script were almost identical (Table 1, Figure 4).

**Figure 5:**
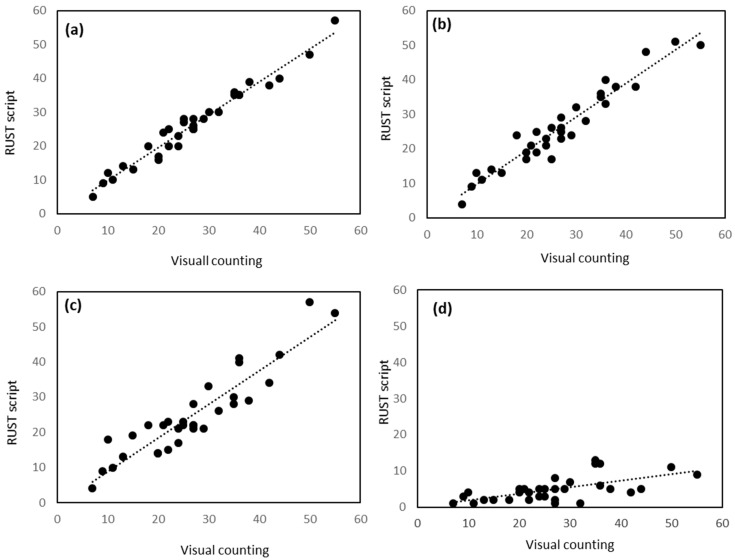
Linear relationship between the number of pustules estimated visually or with RUST under different scenarios. At 1200 ppi with (**a**) or without (**b**) the training step or at 300 ppi with (**c**) or without training step (**d**).

This level of accuracy was obtained with oat leaves scanned at 1200 ppi. To test the accuracy of the script at lower resolutions and the suitability of the training step, a subsample was evaluated visually and with RUST under different conditions (Figure 5). At 1200 ppi, the Lin’s concordance coefficient obtained with the subsample was similar to that obtained with the complete set of samples, confirming the accuracy of the script (Table 1). At this resolution, the evaluation performed without the training step and with default settings slightly reduced the Lin’s concordance coefficient, albeit it remained above 0.96 (*p* < 0.001, Table 1, Figure 5b). Evaluation of lower resolution images with the training step (i.e., 300 ppi, Figure 5c) showed high correlation between visual and RUST values. However, accuracy was slightly reduced compared to the assessment performed at higher resolutions (Figure 5a,b) as observed by the reduction in the Lin’s concordance coefficient to 0.90 (*p* < 0.001). At this lower resolution, the training step was crucial for script accuracy, as Lin’s concordance correlation coefficient was drastically reduced when training was not performed (Figure 5d). It is worth mentioning that, although the Lin’s concordance coefficient decreased drastically, the Pearson correlation coefficient remained above 0.60. This indicates that even without training the correlation between the two variables was still high, although, skipping the training step led to a considerable underestimation of the number of pustules.

Variation coefficients for repeated evaluations of the same leaf were overall low with a mean of 5.0%, with a minimum of 1.8% and a maximum of 7.8%, indicating a low variability of the measurements.

## 3. Discussion

Digital imaging is increasing our analyzing capacity in many agronomic research fields, such as precision agriculture or phenotypic platforms. This enables evaluation of a wide range of parameters through visible and non-visible wavelengths that facilitate our understanding of many physiological processes [38,39]. In the field of disease resistance, digital imaging has sped up the breeding of resistance crops. For instance, analysis of more than 20,000 scanned leaves of wheat infected by *Septoria tritici* blotch showed that resistance mechanisms controlling host damage and pathogen reproduction were under separate genetic control, which open possibilities to combine these mechanisms in new, highly resistant cultivars to this devastating disease [26]. This highlights the high potential of this approach to breed for plant disease resistance.

Although not all research laboratories have access to complex imaging platforms, modern digital cameras and scanners are relatively low cost and available in most laboratories. However, this is not the case with the often expensive software required to analyze images, such as Assess [30], one of the discipline standard systems. Recent applications, such as Leaf Doctor [40] or Bio Leaf [41] are more intuitive and freely accessible, but are less automated and are not specific for evaluating rust infection since they do not evaluate infection frequency. Accurate evaluation of rust diseases, compared to other fungal diseases, is particularly time-consuming and tedious. This is because the commonly used disease severity, evaluated as the percentage of diseased tissue, is not the most appropriate parameter for rust evaluation. Periodical counting of pustule number over the infection process is necessary to determine infection frequency, latency period, and the area under the disease progress curve (AUDPC) [42].

We have developed RUST script as an open source tool that may ease and speed up the rust disease assessment process. RUST users can maintain complete control over the different steps of the process or perform the analysis fully automatically. For most applications, the semi-automated modes (mode 2 or 3) are recommended, since users control the different steps of the analysis without greatly compromising its speed, requiring, on average, 2 min to process 10 images. This is possible due to the automatic and sequential image opening, allowing a lower processing time than that reported for Leaf Doctor or Assess [40]. The fully automated mode (mode 1) is even faster than modes 2 and 3. However, it requires individually scanned/photographed leaves with black or well-outlined background and the previous verification of the suitability of the default color transformation settings. While other applications, such as Leaf Doctor, necessarily require black backgrounds, RUST allows the user to manually delineate the leaf or evaluation area when the background is not sufficiently distinguished from the leaf, for instance for images taken in the field. However, correct recording and labeling of each image is still necessary. This is an important challenge for image analysis approaches and hence, future improvement of the method should aim the automatic image acquisition in the field.

Besides the different automation modes, the script also includes the possibility to calibrate and train the system. Calibration is required to obtain the infection frequency as number of pustules per cm square. This allows comparison of the results between experiments and laboratories regardless the area evaluated or image resolution. This step takes only 15 s once the image with the 1 cm^2^ square shape is uploaded. Training of the system is also an optional step. According to our results, training is not necessary with high-resolution images but it is strongly recommended for lower resolution images. In a previous work, Steddom et al. [43] did not found differences in the analysis of images with different resolutions. This is probably because they analyzed disease severity (i.e., percentage of necrotic leaf area) and did not count individual pustules. Counting of pustules is the most delicate step in the script since the software need to discriminate pustules from any other small spots including the urediniospores that come off from the pustule as a fine powder on the leaf. The addition of these very small dots within the total necrotic area is not a big issue in the measurement of the percentage of diseased tissue, taking into account the larger total values, as observed by Steddom et al. [43] but it is an important issue for counting pustules. To avoid counting these spots erroneously as small pustules, the RUST script identifies a pustule if its size is higher than 50 pixels (default parameter) or than the threshold established from a well-formed pustule during the training. The default size was selected, as it is appropriate for most images of relatively high-resolution, which are usual in modern devices. If training is not performed on lower resolution images, this default threshold underestimates the number of pustules, as we observed. The training step is therefore recommended in all cases since it takes less than 1 min and increases accuracy. When the calibration and training steps were performed, the reliability and accuracy obtained by RUST was very high and comparable to that obtained by previous work. For instance, Ganthaler et al. [44] reported coefficients of determination between 0.87 (natural background) and 0.95 (black background) when they compared the evaluation of the distinct yellow discoloration of rust attacked needles of spruce forest by image analysis and conventional methods. Similarly, Bock et al. [45] reported bias correction factors between 0.93 and 0.99 in their comparison of the number of citrus canker lesions on grapefruit leaves estimated with Assess or by visual ratings, indicating that image analysis was more reliable when repeated compared to visual raters.

An additional advantage of RUST is the wizard-like process in which all modes have been built. This means that user is guided through the evaluation process at all times, allowing complete evaluation of a set of images stored within a folder with automatic opening of the next images upon automatic storage of the results of the previous one, which save a considerable time for users.

RUST has been developed essentially to evaluate cereal leaves. Accordingly, the color transformation step is based on the blue projections. However, we have implemented other chrominance components in order to extend the applicability of the script. These other chrominance components have proven useful, for instance, in the evaluation of leaves of legumes such as lentils, faba beans, or peas. This will speed up the analysis of infection frequency and other rust disease parameters such as latency period or the AUDPC, which are time-consuming and tedious. The script has been developed for the accurate counting of rust pustules. It also measures the average pustule size and the percentage of diseased tissues as additional disease severity parameters. In addition, its application is not limited to the analysis of diseased leaves, and it can be used, for instance, for automatic colony counting in Petri dishes or other similar applications.

## 4. Materials and Methods

### 4.1. Plant Material and Inoculation

A set of 80 oat (*Avena sativa*) genotypes ranging in rust susceptibility (Sánchez-Martín et al. 2012), provided by “Centro de Recursos Fitogenéticos”, INIA, Madrid, Spain were used for validation of the script. Seedlings were grown individually in 50 mL tubes filled with peat:sand (2:1) in a growth chamber at 20 °C, 65% relative humidity, and under 12 h photoperiod with 250 μmol m^−2^ sec^−1^ photon flux density supplied with high-output white fluorescent tubes. All experiments used fully expanded first-formed leaves of 11 day-old plants (their second-formed leaf was unrolling).

*Puccinia coronata* f. sp. *avenae* isolate Co17, derived from a bulk population collected at a naturally infected oat field in Córdoba (Spain) in 2017 and stored at Institute for Sustainable Agriculture (IAS-CSIC), was used in all experiments. Urediniospores were multiplied on plants of cvs. Cory and Araceli, which were highly susceptible. One day before experimental inoculation, spores were collected and kept overnight in a desiccator.

When plants had the first fully expanded leaf, they were inoculated with urediniospores mixed with pure talcum (1:1, *w/w*) by dusting them over the adaxial surface of first leaves of intact plants to give approximately 30 spores mm^−2^ (verified by counts made from glass slides laid adjacent to leaves). In an additional experiment, leaves were heavily inoculated (approximately 100 spores mm^−2^) in order to test the efficiency of the script under highly sporulating leaf conditions. Homogeneous inoculation was ensured by placing leaves horizontally and inoculating them under a settling tower [46,47]. After inoculation, plants were incubated for 12 h in darkness at 100% relative humidity and 18 °C, and thereafter for a week at 20 °C under a 12 h photoperiod with 250 μmol m^−2^ s^−1^ photon flux density.

### 4.2. Image Recording and Analysis

A database was compiled with images from 892 samples taken by scanning the inoculated leaves on an Epson Perfection V370 Photo Scanner (Seiko Epson Corporation, Barcelona, Spain). The rust inoculated leaves showed a wide range of pustule density to assess the robustness of the evaluation process. Prior image acquisition, leaf segments were attached to a black cardboard previously sprayed with repositionable adhesive. Analytical results of each image were recorded for statistical analysis. Images were processed through the script as describe below without editing. In order to assess the image processing time, the number of seconds required to process a set of 10 randomly selected images was recorded.

### 4.3. Statistical Analysis

Between 6 and 12 leaves (from different plants and inoculation experiments) per genotype were studied. This made a total of 892 samples. Linear regression and correlation analysis were used to estimate the relationship and association, respectively, between the number of pustules counted by the rater (independent variable; x) and the digital count performed using the RUST script (dependent variable; y). Accuracy was calculated using Lin’s concordance correlation coefficient (LCCC). LCCC comprises several statistics that define the accuracy (Cb, generalized bias or bias correction factor) and precision (r, Pearson correlation coefficient) of the measurements and indicates the reproducibility of measurements collected by a new instrument/assay/device with respect to the standard method [35,36]. Reliability was calculated using the coefficient of determination (*R*^2^), which explains the proportion of the variance in the dependent variable that is predictable from the independent variable. Repeatability was estimated by calculating the variation coefficients obtained after repeated evaluation of the same image using RUST. To do this, 10 randomly selected images were evaluated independently five times each. All statistical analyses were performed with SPSS Windows v.26 software.

### 4.4. RUST Script

RUST processes the data through five main steps namely training, calibration, selection of areas, color transformation, and pustule analysis. Training and calibration are required only once at the beginning of the analysis and are optional. Training allows users to define a reference pustule for the set of images to be evaluated (recorded with the same settings, i.e., resolution, distance, etc.). The software will count as a pustule any spot up to 85% smaller than the reference pustule.

Calibration is only necessary if the resolution of the uploaded image is unknown and the user wants to obtain infection frequency (IF) in pustules per cm^2^. Calibration step uses the number of pixels contained in a 1 cm^2^ shape to normalize data and convert the unit of the IF to pustules per cm^2^. The 1 cm^2^ shape needs to be drawn or printed using the available template (Supplementary Figure S1) and recorded with the same settings as the rest of the images to be evaluated. Once the image of the shape is saved in the same folder as the images to be evaluated, calibration takes approximately 15 s.

Selection of the leaf areas can be done automatically or manually. The script accurately identifies the leaf area in black background images. The script first attempts automatic selection of the leaf area by the wand tool, which selects pixels of equal or similar value to threshold pixels that form a contiguous area. The wand tool creates selections that have only a boundary line (inner holes are not excluded from the selection) and users can improve the accuracy by adjusting the “tolerance” threshold. The tolerance bar functions by expanding the selection to those points in the image that show a difference with the pixel value of the point clicked below the specified tolerance value.

The color transformation step is based on the ImageJ plugin “Color Transformer” [48], which converts an RGB color image into a color space represented by a stack. The script performs a YUV transformation of the image. YUV is a color space typically used for color image processing, where Y stands for “luma,” brightness, or lightness, and U/V provides color information. The script retains the stack with the chrominance component “U” (blue projection) when the option “Cereals” is selected in the menu and the stack with chrominance “V” (red projection) when selecting “Others”. These chrominance components resulted in the best results for evaluation of different types of leaves, mainly regarding the contrast between the green of the leaf and the brown-orange color of the pustules.

Pustule counting step is a combination of two processes. Before counting, a threshold range is established to specify and differentiate pustules from background. Users can adjust this threshold for greater accuracy in mode 2 and 3 using a slider bar. Moving the bar, users can increase or decrease the pixels within the threshold range until the transformed image is similar to the original RGB one that remains visible on the screen. In mode 1, the threshold is set to a predetermined value. The analysis of the pixels that form a pustule is based on the ImageJ plugin “Analyze particles”. In this step, the image is scanned until the edge of an object, in this case a pustule, is found. The software then outlines the pustule and measures it, fills it to make it invisible, and resumes scanning until the end of the image is reached. The script specifies the minimum number of pixels counted as a pustule. If training is not performed, this value is set by default to 50, which has been useful for good resolution images (over 600 ppi). When training is performed, this value changes according to the reference pustule as explained above.

## Figures and Tables

**Table 1:** Agreement and reliability parameters of the comparison of infection frequency measured visually or with the RUST script under different conditions. Bias correction factor or generalized bias (Cb), Pearson (r), and Lin’s concordance correlation coefficient (LCCC), regression statistics, and model parameters for linear regression were calculated with SPSS software. The full dataset was obtained from *n* = 892 samples. The effect of training on high-resolution (HR) or low-resolution (LR) images was estimated from a set of *n* = 30 samples.

Samples	C_b_^a^	r ^b^	LCCC ^c^	*R* ^2^ ^d^	Slope	Intercept	*p*
Complete set	0.999	0.989	0.989	0.98	0.99	1.46	<0.001
Training HR	0.998	0.982	0.980	0.96	0.97	0.12	<0.001
No Training HR	0.998	0.964	0.962	0.93	0.97	0.08	<0.001
Training LR	0.986	0.921	0.908	0.84	0.95	0.7	<0.001
No Training LR	0.126	0.619	0.078	0.38	0.18	0.09	<0.001

^a^ Generalized bias or bias correction factor (Cb) measures how far the best-fit line deviates from 45° (measure of accuracy). ^b^ The Pearson correlation coefficient (r) measures how far each observation deviated from the best-fit line (measure of precision). ^c^ Lin’s concordance correlation coefficient (LCCC) combines both measures of precision (r) and accuracy (Cb). ^d^ The coefficient of determination (*R*^2^), explains the proportion of the variance in the dependent variable that is predictable from the independent variable and is a quantitative measure of reliability [35,36,37].

## Supplementary Materials

The following are available online at https://zenodo.org/record/4059114#.X4-zYO1YZPZ. Figure S1. Template for calibration step; Script S2. RUST script for evaluation of rust diseases; Video S3. Video representative of Mode 1, Training, calibration and saving results in csv.format; Video S4. Video representative of Mode 2, selection of area manually and automatically, exporting data to excel file; Video S5. Video representative of Mode 3, and training.
